# Electrostatic Field Modification Enhances the Electrocatalytic Oxygen Evolution Reaction Stability of CoFe_2_O_4_ Catalysts

**DOI:** 10.3390/mi16050491

**Published:** 2025-04-22

**Authors:** Liwen Liang, Jiatong Miao, Xiyuan Feng, Yunlei Zhong, Wei Wang

**Affiliations:** 1 Key Laboratory of Multifunctional Nanomaterials and Smart Systems, Division of Advanced Materials, Suzhou Institute of Nano-Tech and Nano-Bionics, Chinese Academy of Sciences, Suzhou 215123, China; 2 School of Environmental Science and Engineering, Changzhou University, Changzhou 213164, China; 3 School of Microelectronics, Northwestern Polytechnical University, No. 1 Dongxiang Road, Chang’an District, Xi’an 710129, China; fengxy@nwpu.edu.cn

**Keywords:** electrostatic field modification, CoFe_2_O_4_/C catalyst, oxygen evolution reaction stability, oxygen vacancy regulation

## Abstract

Enhancing the stability of oxygen evolution reaction (OER) catalysts is a critical challenge for realizing efficient water splitting. In this work, we introduce an innovative approach by applying an electric field during the annealing of a CoFe_2_O_4_/C catalyst. By controlling the electric field strength (100 mV) and treatment duration (1 h), we achieved dual optimization of the catalyst’s microstructure and electronic environment, resulting in a significant improvement in catalytic stability. The experimental results demonstrate that the electric field-treated catalyst exhibits a reduced overpotential decay (only 0.8 mV) and enhanced stability (retaining 89.1% of its initial activity after 24 h) during extended OER testing. This performance significantly surpasses that of the untreated sample, which showed an overpotential decay of 1.5 mV and retained only 72.5% of its activity after 24 h. X-ray photoelectron spectroscopy (XPS) analysis confirmed that the electric field treatment promoted the formation of oxygen vacancies, substantially improved electron transfer efficiency, and optimized the local electronic environment of Co^2+^/Co^3+^ and Fe^2+^/Fe^3+^, leading to a decrease in charge transfer resistance (Rct) from 58.2 Ω to 42.9 Ω. This study not only presents a novel strategy for modulating catalyst stability via electric fields but also broadens the design concepts for OER catalytic materials by establishing a structure–activity relationship between electric field strength, microstructure, and catalytic performance, ultimately providing a theoretical foundation and experimental guidance for the development of highly efficient and stable water splitting catalysts.

## 1. Introduction

The oxygen evolution reaction (OER) is critical in electrolytic water hydrogen production, metal–air batteries, and other emerging energy conversion technologies, with its efficiency and stability directly impacting overall energy conversion performance [1,2,3]. However, the OER is a complex, multi-step reaction involving electron transfer and adsorption/desorption processes, typically associated with high overpotentials and rapid electrocatalyst degradation [4,5,6]. While new catalytic materials have been continuously developed in recent years, achieving substantial improvement in the long-term stability of catalysts while maintaining high catalytic activity remains a significant bottleneck limiting the practical application of OER.

Oxygen Evolution Reaction (OER) is a crucial reaction in energy conversion systems such as water splitting and metal–air batteries. However, due to the multi-electron transfer process involved in OER, the reaction kinetics are slow, and the catalyst is prone to degradation, which severely limits its practical application. Consequently, the development of highly efficient and durable OER catalysts has emerged as a critical research frontier in contemporary electrocatalysis. Transition metal oxides, especially spinel-type (MFe_2_O_4_, M = Co, Ni, Mn, etc.) catalysts, have received extensive attention due to their rich redox centers, excellent electrochemical activity, and low cost [7,8,9,10,11]. However, these catalysts often face problems such as structural collapse, active site dissolution, and changes in surface chemical environment during long-term operation, leading to a decrease in their stability [12,13,14]. Therefore, how to effectively improve the stability of OER catalysts and extend their service life is one of the core challenges in current research.

Currently, researchers primarily focus on controlling material composition, optimizing microstructure, and adjusting electronic states to improve catalyst stability [15,16,17]. For example, transition metal oxide-based catalysts are a focal point in OER research due to their diverse oxidation states and low cost. Multi-metal oxide systems composed of elements such as iron, cobalt, and nickel, for instance, offer abundant active sites for the reaction due to their unique crystal structures [18,19,20]. However, during long-term electrochemical operation, these catalysts often exhibit performance degradation due to structural reconstruction, oxygen vacancy changes, and metal dissolution. To address this, strategies such as electrostatic field polarization processing [21], in situ characterization [22], and defect engineering [23] are gaining attention. Electric field treatment can improve crystallinity, optimize the long-range ordered structure of the catalyst, and regulate the surface oxygen vacancy and metal oxidation state distribution, thereby enhancing catalyst durability to some extent [24,25].

In this work, we employed a constant-current electric field treatment to modulate the CoFe_2_O_4_/C composite catalyst and systematically investigate the electric field’s impact on its oxygen evolution reaction (OER) stability. Electrochemical active surface area (ECSA) and electrochemical impedance spectroscopy (EIS) measurements revealed that the catalyst’s active surface area decreased following the electric field treatment. However, its interfacial charge transfer kinetics improved, as evidenced by a lower charge transfer resistance (Rct). Concurrently, linear sweep voltammetry (LSV) cycling and chronoamperometry (i-t) tests both demonstrated that the electric field treatment significantly reduced the performance degradation of the catalyst during extended OER testing. X-ray photoelectron spectroscopy (XPS) analysis further revealed that the electric field-induced increase in oxygen vacancies resulted in a shift of the O 1s binding energy to higher energies, while the cobalt and iron binding energies shifted to lower energies. This indicates that the electric field optimized the catalyst’s electronic environment and enhanced its structural stability.

## 2. Experimental Section

### 2.1. Chemicals

Cobalt nitrate hexahydrate (Co(NO_3_)_2_ 6H_2_O, ≥98.98%), iron(II) sulphate nonahydrate (Fe(NO_3_)_3_ 9H_2_O, ≥98.98%), carbon powder (C, ≥97.9%), ethanol (CH_3_CH_2_OH, 98.88%), nitrogen (N_2_, supplied by Linde Gas, Suzhou, China), and deionized water (DI) were utilized in the synthesis process.

### 2.2. Fabrication of CoFe_2_O_4_

Following the precise weighing of 20 mg of cobalt(II) nitrate hexahydrate (Co(NO_3_)_2_·6H_2_O) and 25 mg of iron(III) nitrate nonahydrate (Fe(NO_3_)_3_·9H_2_O), these were transferred, in a mass ratio of 1:1.25, alongside a suitable quantity of pre-dried carbon black into a clean 100 mL round-bottomed flask. Subsequently, 50 mL of ethanol/water (1:1 *v*/*v*) solution was slowly introduced. The flask was then placed in a water bath sonicator and subjected to continuous sonication at a frequency of 40 kHz and a power of 200 W for 30 min, ensuring the thorough dispersion of the metal salts and the formation of a homogenous suspension. The well-mixed dispersion was then transferred to an oil bath equipped with a magnetic stirrer. The oil bath temperature was set to 100 ± 2 °C, and the mixture was continuously heated and dried under a stirring speed of 600 rpm until the complete evaporation of the solvent, a process requiring approximately 4–6 h. Once the sample was completely dry, the catalyst powder adhering to the inside of the flask was carefully scraped off using a clean spatula. The resulting black solid powder, representing the initial cobalt–iron bimetallic catalyst precursor, was then stored in a desiccator pending subsequent calcination and characterization experiments.

### 2.3. Annealing Treatment

The prepared catalyst underwent an annealing process to optimize its structure and performance. Initially, the catalyst sample was evenly distributed within the isothermal zone of a tubular reactor, ensuring complete contact between the sample and the reactor’s inner wall. High-purity nitrogen gas (purity ≥ 99.999%) served as a protective atmosphere, flowing continuously into the reactor to purge oxygen and other gaseous impurities. Subsequently, the reactor temperature was increased to 350 °C at a ramp rate of 5 °C/min and held at this temperature for 1 h to facilitate heat treatment, relieving residual stress within the catalyst and enhancing its crystallinity. Concurrently, to investigate the impact of an applied electric field on catalyst performance, a separate catalyst sample was subjected to a constant current of 100 mA under identical heat treatment conditions. A constant current generator precisely controlled the current intensity, and the sample was treated at 350 °C for 1 h. Throughout the experiment, the reactor’s internal temperature and gas flow rate were monitored in real time to guarantee the stability and reproducibility of the experimental parameters. Following the treatment, the reactor was cooled to room temperature at a rate of 10 °C/min. Ultimately, two groups of catalyst samples, each treated under different conditions, were obtained and labeled as CoFe_2_O_4_/C (conventional annealing) and CoFe_2_O_4_/C-EF (electric field-assisted annealing), respectively, for subsequent characterization and performance evaluation.

### 2.4. Materials Characterization

The catalyst was characterized using SEM, XRD, and XPS. Scanning electron microscopy (SEM) with a Hitachi S-4800 was used to obtain morphological images of the catalyst. X-ray diffraction (XRD) was performed using a D8 Advance system. X-ray photoelectron spectroscopy (XPS) analysis was conducted using a Thermo Scientific Escalab 250XI, Science, Suzhou, China.

### 2.5. Electrochemical Measurements

All electrochemical measurements, including rotating-disk electrode (RDE) polarization curve (LSV, 5 mV s^−1^, positive scanning), cyclic voltammetry (CV, 5 mV s^−1^, positive scanning), and chronoamperometry measurement, were performed in O_2_-saturated 1 M KOH by a three-electrode cell with an electrochemical workstation (CHI 760E, Chenhua, Shanghai, China). The reference, counter, and working electrodes were an Hg/HgO electrode (1 M KOH), a carbon-rod electrode, and an RDE with a glassy-carbon electrode (0.19625 cm^2^), respectively. All potentials (iR corrections) were calibrated to the reversible hydrogen electrode (RHE) by the following conversion formula (ERHE = E_Hg/HgO_ + 0.9224 V). For electrode preparation, typically, 8 mg of catalysts were dispersed in 500 μL of water/ethanol (3/1 *v*/*v*) solution containing 5 μL of Nafion, and then the mixture was treated by ultrasound for 5 min. Afterwards, catalyst loading was standardized to 0.81 mg/cm^2^ via analytical balance precision (±0.01 mg). Drop-casting uniformity was ensured by depositing 10 μL/cm^2^ of ink onto pre-cleaned electrodes, followed by drying at 60 °C for 10 min. The electrochemically active surface areas (ECSAs) of various CoFe_2_O_4_/C catalysts were determined by double-layer capacitance (Cs) measurement. Additionally, the electrochemical impedance spectra (EIS) were gained at 1600 rpm from 1000 to 0.01 Hz in O_2_-saturated 1 M KOH. To study the oxygen species absorbance on the catalyst surfaces, the oxygen reduction reaction (OER) polarization curves were measured from 1.3 to 1.76 V vs. RHE (5 mV s^−1^, negative scanning) at 1600 rpm.

## 3. Results and Discussion

### 3.1. Morphological Characterization

**Figure 1:**
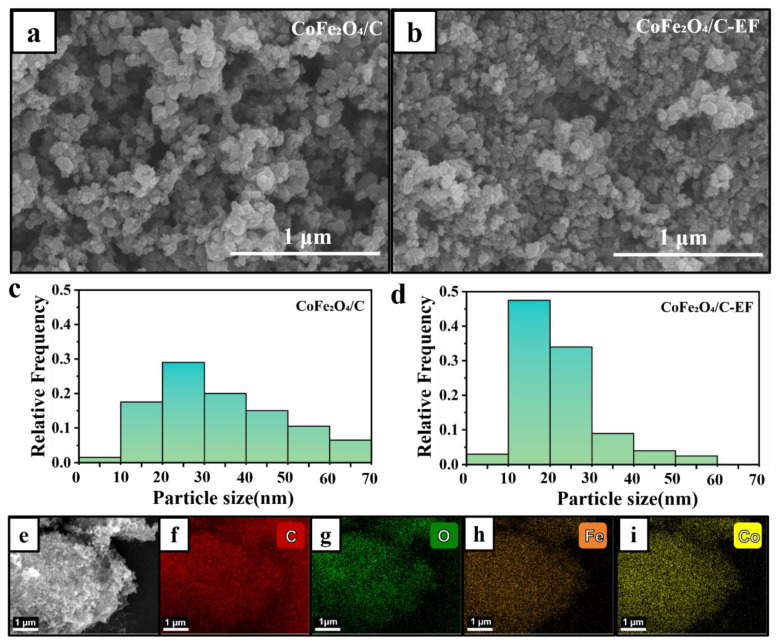
SEM images of (**a**) CoFe_2_O_4_/C, (**b**) CoFe_2_O_4_/C-EF; particle size distribution of (**c**) CoFe_2_O_4_/C, (**d**) CoFe_2_O_4_/C-EF; (**e**–**i**) EDX element mapping images of CoFe_2_O_4_/C-EF.

The surface morphology of the synthesized CoFe_2_O_4_/C catalyst was investigated via SEM imaging. Figure 1a,b illustrate the morphology of the catalyst annealed without (CoFe_2_O_4_/C) and with an applied electric field (CoFe_2_O_4_/C-EF), respectively. Evidently, all catalysts consist of irregularly shaped spherical particles with varying dimensions, resembling aggregated coral. A comparison of SEM morphologies between CoFe_2_O_4_/C and CoFe_2_O_4_/C-EF reveals that the electric field-treated catalysts exhibit more pronounced aggregation. This heightened aggregation could be attributed to the generation of local electric field gradients and polarization effects induced by the applied electric field. These effects enhance the attraction between crystal particles, promoting their aggregation or agglomeration. Furthermore, the electric field might intensify the directional aggregation of crystal nuclei, causing the initially dispersed crystals to cluster together, thereby decreasing their dispersion [24]. As shown in Figure 1c,d, the particle size distribution for the catalyst without an applied electric field is predominantly within the 20–30 nm range, whereas the particle size distribution for the catalyst treated with an electric field is largely within the 10–20 nm range. The reduced size of CoFe_2_O_4_/C-EF nanoparticles suggests that the introduction of an electric field influences the crystal growth kinetics, inhibiting grain growth and leading to a decrease in crystal crystallinity. Figure 1e–i present EDX mapping of the catalyst treated with an electric field, clearly demonstrating the presence of Co, Fe, and O elements, uniformly distributed within the carbon region.

### 3.2. Structural Characterization

For materials not subjected to electric field annealing, detailed X-ray diffraction (XRD) analysis revealed diffraction peaks at 35.4°, 43.2°, and 62.5°, which closely correspond to the (311), (400), and (440) planes of the cubic spinel structure of CoFe_2_O_4_ (JCPDS: 00-001-1121). Importantly, a comprehensive examination of the entire XRD pattern showed no diffraction peaks associated with other impurities, strongly suggesting the successful formation of pure spinel ferrite nanoparticles [26,27,28]. When examining the CoFe_2_O_4_/C-EF, the XRD patterns displayed both similarities and differences. The XRD peaks of CoFe_2_O_4_/C-EF are less sharp than those of CoFe_2_O_4_/C. This variation may be attributed to the application of an electric field during the annealing process, which potentially reduces the crystallinity of the material. The introduction of an electric field can influence the kinetics of crystal growth, inhibiting grain enlargement and leading to decreased crystallinity. Electric field treatment significantly modulates the crystal structure and crystallization behavior of CoFe_2_O_4_/C materials, providing a foundation for further investigating the underlying mechanisms of electric field effects on material properties.

**Figure 2:**
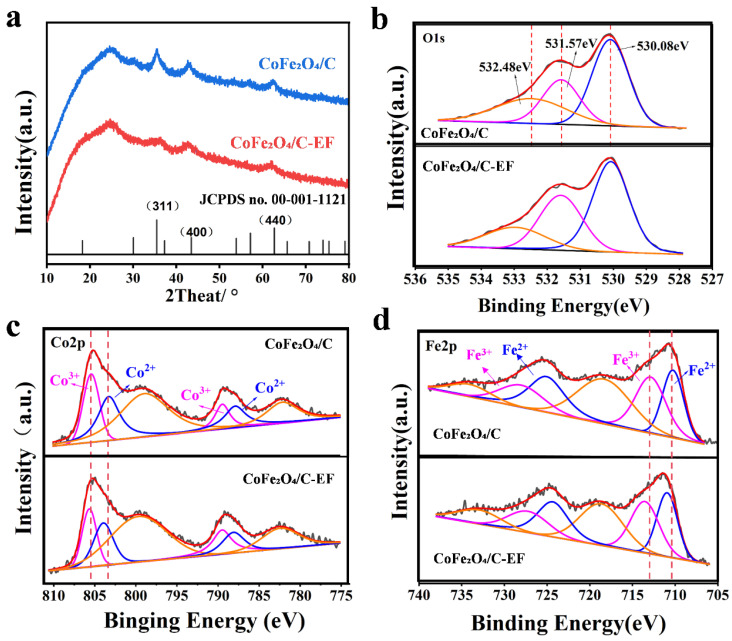
(**a**) XRD patterns of CoFe_2_O_4_/C and of CoFe_2_O_4_/C-EF; XPS spectra, (**b**) O 1s, (**c**) Co 2p, and (**d**) Fe 2p of CoFe_2_O_4_/C and CoFe2O_4_/C-EF.

The O 1s spectra (Figure 2b) obtained from the CoFe_2_O_4_/C catalyst were fitted using three peaks, designated as O_latt_, O_def_, and O_ads_, which were located at 530.08 eV, 531.57 eV, and 532.48 eV, respectively. The O_latt_ peak was attributed to metal–oxygen (Co-O and Fe-O) bonds. The O_def_ peak corresponded to oxygen defects/vacancies, and the O_ads_ peak represented hydroxyl (OH) groups adsorbed on the catalyst surface [29]. The electric field treatment clearly had a notable impact on the O 1s component. Specifically, an increase in O_def_ was observed for CoFe_2_O_4_/C, indicating a higher concentration of defect sites in the CoFe_2_O_4_/C-EF compared to the CoFe_2_O_4_/C catalyst. Through calculating the peak area ratio of the oxygen element, it was found that the oxygen vacancy content of CoFe_2_O_4_/C-EF increased by 9.03% compared to that of CoFe_2_O_4_/C. This increase directly correlates with the reduced Rct value (from 58.2 Ω to 42.9 Ω), as oxygen vacancies enhance electron transfer efficiency by introducing localized defect states. The result aligns with the findings from SEM and XRD analyses. The reason for this phenomenon may be that under the action of an electric field, oxygen ions in the crystal may undergo directional migration, especially under high-temperature annealing conditions, where this migration is more significant. Under the influence of the electric field, oxygen ions within the crystal lattice likely underwent directional migration, especially pronounced under high-temperature annealing. This movement of oxygen ions potentially led to the formation and accumulation of oxygen vacancies within the lattice structure. Simultaneously, owing to thermal-electric synergy, the Joule heating effect induced by the electric field may have elevated the local temperature and facilitated oxygen desorption, thus augmenting the oxygen vacancy concentration. This is associated with electric field-induced oxygen vacancy formation. Concurrently, the generation of oxygen vacancies modified the surrounding chemical environment, resulting in an increased binding energy of the oxygen 1s electrons, which manifested as a peak shift towards higher binding energies in the XPS spectra. The increased oxygen vacancy content likely enhanced the surface redox properties of the catalyst.

The effect of an electric field on the oxidation state of the active CoFe_2_O_4_ component was investigated. High-resolution Co 2p and Fe 2p spectra were acquired from the catalysts and are presented in Figure 2c,d. The Co 2p spectra for all catalysts exhibited two spin-orbit doublets, Co 2p3/2 and Co 2p1/2, and satellite features (Sat.) at higher binding energies for both the Co 2p3/2 and Co 2p1/2 peaks. For the CoFe_2_O_4_/C catalyst annealed without an applied electric field, the Co 2p3/2 peak was deconvoluted into two peaks at 779.60 eV (Co^2+^ in octahedral sites) and 781.5 eV (Co^3+^ in tetrahedral sites). Similar fitting was performed for the Co 2p3/2 peaks in catalysts annealed with an applied electric field [30,31]. This indicates that cobalt exists in two oxidation states, Co^2+^ and Co^3+^. However, the Co peaks for the materials annealed under the influence of an electric field shifted toward lower binding energies, implying that the application of the electric field during annealing significantly affected the crystal structure of the cobalt ferrite spinel, thus altering the local electronic environment of cobalt. Concurrently, the Co^2+^ and Co^3+^ content in CoFe_2_O_4_/C and CoFe_2_O_4_/C-EF were compared. The ratio of Co^2+^ to Co^3+^ was higher for CoFe_2_O_4_/C-EF (Co^2+^:Co^3+^ = 1:0.97) than for CoFe_2_O_4_/C (Co^2+^:Co^3+^ = 1:1.20). The electric field is thought to accelerate atomic diffusion and lattice rearrangement, improving the long-range order within the crystal structure. This results in a reduction in internal defects and grain boundaries, placing cobalt ions in a more homogeneous and stable chemical environment. In this environment, the more balanced local charge distribution reduces the effective nuclear charge shielding of the cobalt atoms, leading to the observed decrease in binding energy measured by XPS. Simultaneously, the electric field may rearrange the bonding between oxygen and the metals, modifying the coordination environment around the cobalt. This change could enhance electron delocalization, increasing the electron cloud density of the cobalt atoms and further contributing to the observed shift of the cobalt peaks to lower binding energies in the XPS spectra.

All studied catalysts exhibited Fe 2p spectra with two doublets, Fe 2p3/2 (711.3 eV) and Fe 2p1/2 (725.6 eV), as well as two satellites associated with Fe 2p3/2 and Fe 2p1/2, regardless of whether an electric field was applied (Figure 2d). The presence of satellite peaks at binding energies (B.E.) of approximately 717.5 and 732.2 eV corroborated the iron oxidation state observed in the X-ray diffraction (XRD) results (Figure 2a). Furthermore, similar to the trend observed for Co, the Fe 2p peak positions for samples annealed with an applied electric field shifted towards lower binding energies compared to those annealed without an electric field. The observed phenomenon is primarily attributed to changes in the iron oxidation state. Research indicates that the Fe^2+^/Fe^3+^ ratio is maintained at 1.02:1 in the CoFe_2_O_4_/C system. However, this ratio increases to 1.06:1 in the CoFe_2_O_4_/C-EF system. The underlying cause of this phenomenon is consistent with the cause of the Co 2p peak shift.

The experimental results indicate that, following electric field treatment, Fe ions display a marked tendency to lose electrons, while Co ions exhibit a pronounced ability to capture them. This electron transfer characteristic facilitates the creation of oxygen vacancies at Co sites and concurrently increases the Fe^3+^ concentration, consequently boosting hydroxyl group adsorption. Mechanistically, the application of an electric field in a nitrogen atmosphere effectively promotes the migration of lattice oxygen, leading to a greater density of oxygen vacancy defects within the material [32,33].

### 3.3. Electrocatalytic Performance

**Figure 3:**
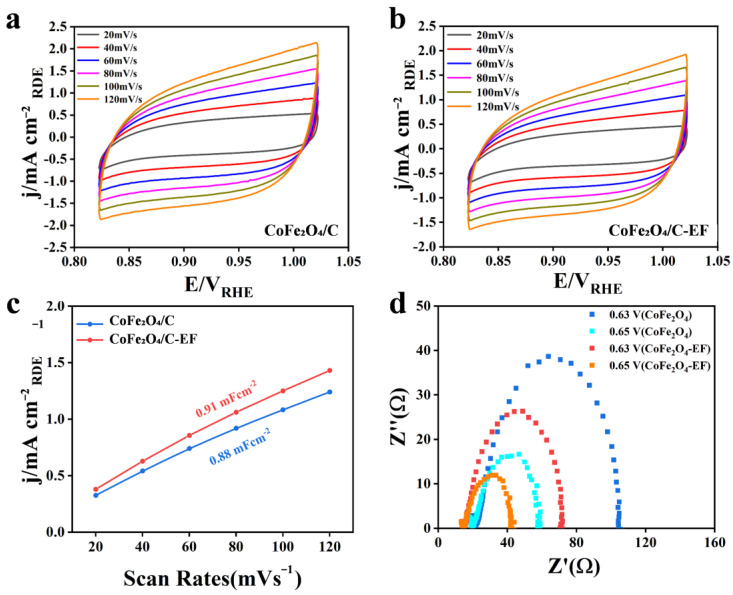
(**a**) ECSA of CoFe_2_O_4_/C; (**b**) ECSA of CoFe_2_O_4_/C-EF; (**c**) C_dl_ plots of CoFe_2_O_4_/C and CoFe_2_O_4_/C-EF; (**d**) Nyquist plots at CoFe_2_O_4_/C and CoFe_2_O_4_/C-EF.

The effect of electric field treatment on the electrochemical properties of CoFe_2_O_4_/C composites was systematically studied using electrochemical active surface area (ECSA) and electrochemical impedance spectroscopy (EIS). Figure 3a and Figure 3b respectively depict the ECSA of CoFe_2_O_4_/C and CoFe_2_O_4_/C-EF. As illustrated in Figure 3c, the double-layer capacitance (C_dl_) value for CoFe_2_O_4_/C-EF increases to 0.91 mF cm^−2^, suggesting a wealth of active sites on the material’s surface. This increase in the active surface area can be attributed to a reduction in crystallinity induced by the electric field: the applied electric field leads to smaller CoFe_2_O_4_ nanoparticle sizes, consequently increasing the material’s specific surface area and the number of readily accessible active sites. To further confirm the effect of electric field treatment on the material’s electrochemical properties, EIS spectra were acquired at different voltages (Figure 3d). The results revealed that the electric field-treated CoFe_2_O_4_/C exhibited a smaller charge transfer resistance (Rct) and faster interfacial charge transfer kinetics, possibly because the electric field treatment optimized the material’s electronic structure and interfacial properties, thereby facilitating electron conduction and ion diffusion. Importantly, although the electric field treatment led to a decrease in active area, its improvement of the material’s overall electrochemical performance was more pronounced, suggesting that electric field treatment plays a crucial role in regulating the electrochemical properties of the material. These findings offer new insights into the influence mechanism of electric field treatment on the electrochemical properties of transition metal oxide/carbon composites and provide effective strategies for optimizing the electrochemical performance of such materials.

### 3.4. Stability Assessment

To comprehensively evaluate the electrocatalytic stability of the catalysts, we conducted the systematic characterization of catalysts under two different treatment conditions using a standard three-electrode setup in a 1 M KOH electrolyte. During testing, we employed a CHI 760E electrochemical workstation to acquire 10 consecutive linear sweep voltammetry (LSV) curves for each catalyst group at a scan rate of 5 mV/s within a potential range of 1.0–1.8 V (vs. RHE). Comparative analysis revealed that the CoFe_2_O_4_/C-EF exhibited a significant enhancement in electrochemical stability. The overpotential difference at 10 mA/cm^2^ between the 10th LSV curve and the 1st curve was only 0.8 mV for CoFe_2_O_4_/C-EF, compared to 1.5 mV for CoFe_2_O_4_/C catalysts without electric field treatment.

**Figure 4:**
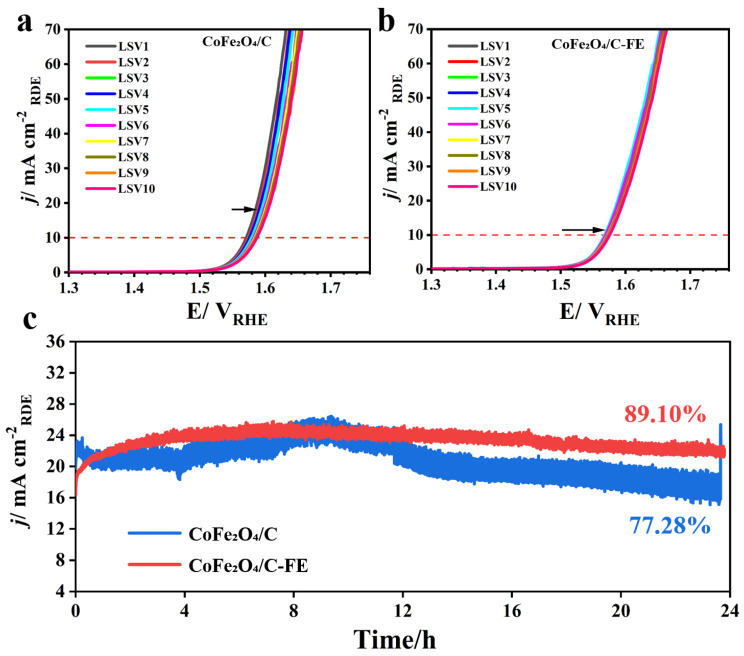
(**a**) LSV curves of CoFe_2_O_4_/C; (**b**) LSV curves of CoFe_2_O_4_/C-EF; the arrows denote the direction along the horizontal axis in which the LSV curve shifted during the tests from the 1st to the 10th in (a) and(b); (**c**) Current–time (i–t) chronoamperometric responses for OER on the CoFe_2_O_4_/C and CoFe_2_O_4_/C-EF at 0.60 V in O_2_-saturated 1 M KOH at a rotating speed of 1600 rpm.

Besides the superior electrocatalytic performance of OER electrocatalysts, durability is also crucial. We assessed the OER stability of CoFe_2_O_4_/C, with and without an applied electric field during annealing, using chronoamperometry in O_2_-saturated media at a rotation rate of 1600 rpm. As shown in Figure 4c, the electric field-treated CoFe_2_O_4_/C maintained 89.1% of its electrocatalytic performance after 24 h of continuous operation at a constant voltage of 0.60 V. In contrast, the OER electrocatalytic performance of the CoFe_2_O_4_/C without electric field treatment was only 77.28% after 24 h of operation at the same voltage. This result indicates that moderate particle aggregation contributes to enhanced structural stability, reducing particle dissolution or loss and consequently extending the catalyst’s lifespan.

Electric field treatment leads to an increase in the material’s active surface area and enhanced charge transfer. This is likely due to the treatment causing a reduction in particle size and an increase in oxygen vacancies, changes that are more conducive to adsorption and activation.

## 4. Conclusions

This study systematically examines the impact of electric field treatment on the oxygen evolution reaction (OER) stability of CoFe_2_O_4_/C composite catalysts, elucidating the underlying regulatory mechanism. Electrochemical active surface area (ECSA) and electrochemical impedance spectroscopy (EIS) measurements indicated that while the electric field-treated catalyst exhibited a slight reduction in active surface area, it displayed significantly improved interfacial charge transfer kinetics, characterized by a lower charge transfer resistance (Rct), thereby facilitating efficient OER performance.

Long-term OER stability tests demonstrated that electric field treatment markedly enhanced the catalyst’s durability. The catalyst treated with an electric field retained 89.1% of its initial current density after 24 h of continuous operation under stirring at 1600 rpm, whereas the untreated catalyst retained only 77.28%. X-ray photoelectron spectroscopy (XPS) analysis further revealed that the electric field induced the formation of oxygen vacancies, leading to a shift in the oxygen 1s binding energy towards higher energy levels, while the binding energies of cobalt and iron shifted towards lower energy levels. These findings suggest that the electric field optimized the electronic environment of the catalyst, thereby improving its structural stability.

In conclusion, electric field treatment effectively enhances the OER stability of the catalyst by modulating its microstructure and electronic environment. This approach provides a novel strategy for the optimized design of transition metal oxide catalysts and lays the groundwork for their application in renewable energy conversion technologies.

## Data Availability

The data that support the findings of this study are available from the corresponding author, upon reasonable request.
